# GC-MS analysis, antimicrobial, antioxidant, antilipoxygenase and cytotoxic activities of *Jacaranda mimosifolia* methanol leaf extracts and fractions

**DOI:** 10.1371/journal.pone.0236319

**Published:** 2020-07-29

**Authors:** Rabia Naz, Thomas H. Roberts, Asghari Bano, Asia Nosheen, Humaira Yasmin, Muhammad Nadeem Hassan, Rumana Keyani, Sami Ullah, Wajiha Khan, Zahid Anwar

**Affiliations:** 1 Department of Biosciences, COMSATS University, Islamabad, Pakistan; 2 Plant Breeding Institute, Sydney Institute of Agriculture, University of Sydney, Sydney, NSW, Australia; 3 Department of Biosciences, University of Wah, Wah Cantt, Pakistan; 4 Department of Botany, University of Peshawar, Peshawar, Pakistan; 5 Department of Biotechnology, COMSATS University Islamabad, Abbotabad Campus, Abbotabad, Pakistan; 6 Department of Computer Science, COMSATS University Islamabad, Vehari Campus, Vehari, Pakistan; Higher Institute of Applied Sciences and Technology of Gabes University of Gabes, TUNISIA

## Abstract

*Jacaranda mimosifolia* trees are grown in frost-free regions globally. The aim of this study was to evaluate the methanol crude extract and various fractions of increasing polarity of *J*. *mimosifolia* leaves for bioactive metabolites, as well as antimicrobial, antioxidant and anticancer activities. The anti-inflammatory potential of the various fractions of *J*. *mimosifolia* leaf extract was studied via the lipoxygenase (LOX) inhibitory assay. Methanol crude extract (ME), derived fractions extracted with chloroform (CF) and ethyl acetate (EAF), and residual aqueous extract (AE) of dried *J*. *mimosifolia* leaves were assayed for polyphenolic compounds, their antioxidant, antimicrobial and lipoxygenase (LOX) inhibitory activities, and anticancer properties. Polyphenolic compounds were determined via HPLC while phytochemicals (total phenolics, flavonoids, tannins and *ortho*-diphenol contents), antioxidant activities (DPPH, hydrogen peroxideperoxide, hydroxyl and superoxide radical anions) and LOX were measured via spectrophotometry. Methanol extracts and various fractions were evaluated for antibacterial activities against *Bacillus subtilis*, *Klebsiella pneumonia*, *Pseudomonas aeruginosa* and *Staphylococcus aureus*. Antifungal potential of the fractions was tested against three species: *Aspergillus flavus*, *Aspergillus fumigatus* and *Fusarium oxysporum*. The highest values for total phenolic content (TPC), total flavonoid content (TFC), flavonols, tannins and *ortho*-diphenols were in the ME, followed by CF > EAF > AE. ME also had the highest antioxidant activity with EC_50_ values 48±1.3, 45±2.4, 42±1.3 and 46±1.3 μg/mL based on the DPPH, hydrogen peroxide, hydroxyl radical and superoxide radical assays, respectively. TPC and TFC showed a significant, strong and positive correlation with the values for each of these antioxidant activities. ME exhibited anti-inflammatory potential based on its LOX inhibitory activity (IC_50_ = 1.3 μg/mL). ME also had the maximum antibacterial and antifungal potential, followed by EAF > CF > AE. Furthermore, ME showed the strongest cytotoxic effect (EC_50_ = 10.7 and 17.3 μg/mL) against human hormone-dependent prostate carcinoma (LnCaP) and human lung carcinoma (LU-1) cell lines, respectively. Bioactive compounds present in leaf methanol extracts of *J*. *mimosifolia* were identified using gas chromatography–mass spectrometry (GC–MS). Fifteen compounds were identified including phenolic and alcoholic compounds, as well as fatty acids. Our results suggest that *J*. *mimosifolia* leaves are a good source of natural products with antioxidant, anti-inflammatory and anti-cancer properties for potential therapeutic, nutraceutical and functional food applications.

## 1. Introduction

Contemporary interest in medicinal plants originates from their extensive use in traditional medicines, especially in developing countries [1]. Many medicinal plant species have been investigated for their bioactive metabolites and antioxidative potential with the aims of (a) finding alternative sources of antibiotics and natural antioxidants for the pharmaceutical and cosmetic industries, (b) promoting health through consumption of products containing natural antioxidants, and (c) identifying ingredients to improve the sensory and nutritional quality of foods and their possible use for the formulation of new food products [2, 3]. Although the toxicity profiles of most medicinal plants have not been thoroughly evaluated, it is often thought that medicines derived from plant products are safer than their synthetic counterparts [4, 5].

Natural antioxidants in the form of their chemical constituents or raw plant extracts are very effective (at least *in vitro*) in preventing harmful conditions instigated by oxidative stress [6, 7]. These antioxidants have the ability to scavenge free radicals, which are usually in the form of reactive oxygen or nitrogen species (ROS/RNS). Oxidative stress may be associated with the appearance of various ailments such as neurological and cardiovascular disorders, as well as cancer [8]. Many studies have reported the positive potential of certain antioxidants to help prevent these diseases [9, 10].

Demand for therapeutic natural products with antioxidant activities capable of lessening the detrimental effects of free radicals has increased [11]. These natural products are reported to have very low toxicities as compared to widely used synthetic antioxidants used in drugs, cosmetics, and food products [12, 13].

Among the chemical constituents of medicinal plants, flavonoids and other phenolic compounds are some of the most important, particularly due to their potential against oxidative stress [14, 15]. These phytochemicals are reported for their antimicrobial potential [16] and anticancer potential; indeed, moremore than 51% of anticancer drugs have been derived from natural sources [17]. Among all the natural compounds, flavonoids and phenolic compounds extracted from various plant species are reported for their cytotoxic potential against several cell lines [18, 19, 20].

Inflammation is a complex biological response of the vascular tissues to a range of stimuli, including irritants, injured cells and pathogens. Injuredinjured or damaged cellscell linked with inflammation have been shown to release arachidonic acid from the cell membrane [21]. Arachidonic acid is then processed via two pathways: the cyclooxygenase (COX) [22] and the lipoxygenase (LOX) pathway [23]. The products of these two pathways are involved in the incidence of many inflammatory diseases including arthritis [24], chronic pain, fever [25], burnsburn, sepsis [26] carcinogenic progressionsprgressions in colorectal cancer [27], and inflammatory bowel disease [28]. The processes linked with inflammatory responses are intricate and usually involve ROS [29]. Hence protection against ROS via the activities of anitioxidant compounds anitioxidants including phenolicsphenolic would provide protection against inflammation.

*Jacaranda mimosifolia* (D. Don) is an ornamental tree belonging to the family *Bignoniaceae*, widely distributed in tropical and subtropical areas of the world. Extracts of various organs of *J*. *mimosifolia* are used in several countries including Bangladesh, India and Pakistan to treat hypertension, ulcers, wounds, diarrhea, dysentery and amoebic infections [30, 31, 32]. In previous phytochemical studies, triterpenes, acetoside, flavonoids, phenylpropanoid derivatives, quinones, anthocyanins and fatty acids have been isolated from the extracts of *J*. *mimosifolia* leaves and flowers [33, 34]. Despite reports on the phytochemicals, antioxidants and antimicrobial potential of *J*. *mimosifolia* extracts, various aspects related to these phytochemicals including polyphenolic compounds, as well as otherother biological activities such as anti-inflammatory and cytotoxic activities remain unexplored. Here we aimed to evaluate the crude methanol extract and various fractions of increasing polarity of *J*. *mimosifolia* leaves forbecause these fractions have not be exploredyet their bioactive metabolites, assaying for antimicrobial, antioxidant and anticancer activities. We also examined the anti-inflammatory potential of the various fractions of *J*. *mimosifolia* leaf extract via the lipoxygenase (LOX) inhibitory assay.

## 2. Materials and methods

### 2.1. Chemicals

Methanol, Folin–Ciocalteu’s reagent, 2,2-diphenyl-1-picrylhydrazyl (DPPH•), quercetin, rutin, gallic acid, ascorbic acid, aluminum chloride, potassium acetate, sodium acetate, hydrogen peroxide, phosphate buffer, nutrient broth, dimethyl sulfoxide (DMSO), Sabouraud dextrose agar (SDA), ethyl acetate, ferric chloride, chloroform, sulfuric acid, hydrochloric acid, benzene, ammonium hydroxide, potassium ferro-cyanide, sodium chloride, ethanol, sodium carbonate_,_ terbinafine, streptomycin, bovine serum albumin, casein, perchloric acid, aspirin, Tris-HCl, and trypsin were purchased from MERCK and Sigma–Aldrich (St. Louis, MO, USA). All reagents and chemicals were of analytical grade.

### 2.2. Collection and processing of plant material for extract preparation

Fresh leaves of *J*. *mimosifolia* were collected in March, 2013 from Quaid-i-Azam University Islamabad, Pakistan. The plant was identified by Prof. Dr. Mir Ajab Khan and was submitted to the National Herbarium, Quaid-i-Azam University Islamabad (voucher specimen 41515). The *J*. *mimosifolia* leaves were washed thoroughly with distilled water and shade-dried for 5 d at room temperature (37ºC). The dried leaves were finely ground using an electric grinder and stored in air-tight containers for further use. The pulverized plant material (1 kg) was extracted twice by soaking with 2 L methanol for 48 h at room temperature. The separated extracts were then filtered through Whatman No. 1 filter paper and the methanol filtrate condensed to dryness using a rotary evaporator at 40°C, yielding 16.3% methanol extract (ME) (i.e. the mass of the dried extract represented 16.3% of the mass of the dry leaf starting material). The dried extract was then stored at 4°C until further use. The methanol residue was further suspended for partitioning with chloroform, ethyl acetate and soluble residual aqueous fractions yielding CF (9.6%), EAF (8.5%) and AE (12.2%), respectively.

### 2.3. Determination of plant extract yield (%)

Yield percentage (w/w) from the dried extracts was calculated as:

Yield(%)=(W1×100)÷W2

where W1 is the dry weight of extract after evaporating the solvent and W2 is the weight of the soaked leaf powder.

### 2.4. Preliminary phytochemical analysis

Phytochemical analysis of the *J*. *mimosifolia* extract and fractions was performed to detect the presence of different classes of secondary compounds, including alkaloids, phenolics, flavonoids, tannins, saponins, terpenes, phlobatannins and coumarins [35, 36].

### 2.5. Quantitative analysis of phytochemicals

#### 2.5.1. Total Phenolic Content (TPC)

TPC was determined using the Folin–Ciocalteu colorimetric method [37] with some modifications. An aliquot (0.3 mL) of leaf extract was mixed with Folin-Ciocalteu phenol reagent (2.25 mL). After 5 min, 6% sodium carbonate (2.25 mL) was added and the mixture allowed to stand at room temperature for 90 min. The absorbance was measured at 725 nm in a spectrophotometer (HITACHI Model U-1100). A calibration curve for gallic acid in the range of 200–800 µg/mL was prepared in the same manner and results were expressed as mg gallic acid equivalent (GAE) per gram of extract. The experimentwas performed in triplicate.

#### 2.5.2. Total Flavonoid Content (TFC)

TFC was determined using the aluminium colorimetric method [38, 39] with some modifications using quercetin (20–80 µg/ mL) as the standard. Leaf extract (0.5 mL) and standard (0.5 mL) were placed in separate test tubes and 10% aluminum chloride (0.1 mL), 1 M potassium acetate (0.1 mL), 80% methanol (1.5 mL) and distilled water (2.8 mL) added and mixed. A blank was prepared in the same manner but 0.5 mL of distilled water was used instead of the sample or standard. All tubes were incubated at room temperature for 30 min and the absorbance was read at 415 nm. The concentration of flavonoid was expressed as mg quercetin equivalent (QE) per gram of extract. Each experiment was performed in triplicate.

#### 2.5.3. Total flavonol content

Total flavonol content was determined following the aluminum chloride colorimetric method [40, 41] with some modifications. A calibration curve for quercetin in the range 200–800 µg/mL was prepared. Extract (1 mL) and standard (1 mL) were placed in separate test tubes to which 2% aluminum chloride (1 mL) and 5% sodium acetate (3 mL) were added and mixed. The mixture was then centrifuged at 3000 rpm for 20 min to obtain a clear solution. The absorbance was read at 440 nm and the results expressed as mg quercetin equivalent (QE) per gram of extract. The experiment was conducted in triplicate.

#### 2.5.4. Total condensed tannins

Total condensed tannins (proanthocyanidins) were determined according to the method of Sun et al. [42]. To 50 μL of diluted sample, 3 mL of 4% vanillin solution in methanol and 1.5 mL of concentrated HCl were added. The mixture was allowed to stand for 15 min, and absorbance was measured at 500 nm against methanol as a blank. Total condensed tannins was expressed as mg catechin/g DW. The experiment was performed in triplicate.

#### 2.5.5. Ortho-diphenol content

*Ortho*-diphenol content was determined by the method described by Tover et al. [43]. Sodium molybdate (1 mL) was added to diluted aliquots of the methanolic extracts and the various fractions, mixed and the absorbance measured at 370 nm. *Ortho*-diphenol content was expressed as mg gallic acid equivalents (GAE)/100 g DW. The experiment was conducted in triplicate.

### 2.6. HPLC analysis of major polyphenolic compounds

Separation of polyphenolic compounds was carried out using a ZORBAXZO ECLIPSE, XDB-C18, (5 μm; 4.6 × 150 mm) column (Agilent USA) with a UV-VIS Spectra-Focus detector. Mobile phase A was acetonitrile:methanol:water:acetic acid (5:10:85:1) and mobile phase B was acetonitrile:methanol:acetic acid (40:60:1). The injection volume was 30 μL and the flow rate was 0.8 mL/min. A concentration gradient profile of 0 to 50% B for 0–20 min, 50 to 100% for 20–25 min and 100% B for 40 min was followed. The system was washed after each run and reconditioned for 10 min before analysis of the next sample. Individual standard solutions of polyphenolic compounds and plant extract solutions were prepared at a concentration of 1.0 and 10 mg/mL, respectively. Samples were filtered through 0.45-μm membrane filters. Quantitative identification of the various *J*. *mimosifolia* extract fractions was performed against eighte reference standards; i.e., apigenin, caffeic acid, catechin, gallic acid, kaempferol, isoquercetin, rutin and *trans*-cinnamic acid (Sigma-Aldrich, USA), which were detected at appropriate wavelengths (257, 279, 325 and 368 nm). Polyphenolic compounds were identified and quantified in the samples by comparing their retention times with those of standards used and standard calibration curves, respectively.

### 2.7. Antioxidant activities

#### 2.7.1. DPPH radical scavenging activity

Antioxidant activities of methanol extracts and their various fractions at 50–250 µg/mL were determined via the DPPH radical scavenging system using gallic acid and ascorbic acid as positive controls. The decrease in absorbance was measured at 517 nm.

$$\text{DPPH}\;\text{sacavanging}\;\text{effect}\;\left(\text{\%}\right)=\left[\frac{\text{A}0-\text{A}1}{\text{A}0}\right]\times 100$$ (1)

where A0 is the absorbance of the control reaction and A1 is the absorbance in the presence of the sample [44].

EC50, which is the concentration (μg/mL) of sample required for 50% scavenging of DPPH, was calculated from the graph plotted for scavenging percentage against the sample concentration. Gallic acid and ascorbic acid were used as positive controls in these assays. All samples were analyzed in triplicates.

#### 2.7.2. Hydrogen peroxide (H_2_O_2_) scavenging activity

The ability of the extracts to scavenge hydrogen peroxide was determined according to Ruch et al. [45]. Absorbance of the hydrogen peroxide activity of methanol extracts and their different fractions at 50–250 µg/mL was recorded at 230 nm. Hydrogen peroxide scavenging ability was calculated using the following equation:

$$\text{Hydrogen}\;\text{peroxide}\;\text{scavenging}\;\text{activity}\;\left(\text{\%}\right)=\left[\frac{\text{A}0-\text{A}1}{\text{A}0}\right]\times 100$$ (2)

where A0 is the absorbance of the control and A1 is the absorbance of the sample.

EC50, which is the concentration (μg/mL) of sample required for 50% scavenging of H_2_O_2_, was calculated from the graph plotted for scavenging percentage against the sample concentration. Gallic acid, and ascorbic acid were used as positive controls in these assays. Each experiment was conducted in triplicate.

#### 2.7.3. Hydroxyl radical scavenging activity

Hydroxyl radical scavenging activity was measured by the ability of the different extracts and fractions of *J*. *mimosifolia* leaves to scavenge the hydroxyl radicals generated by the Fe^3+-^ascorbate-EDTA-H_2_O_2_ system (Fenton reaction; [46]). The reaction mixture contained 500 μL of 2-deoxyribose (2.8 mM) in phosphate buffer (50 mM, pH 7.4), 200 μL of premixed ferric chloride (100 mM) and EDTA (100 mM) solution (1:1; v/v), 100 μL of H_2_O_2_ (200 mM) with or without the extract solution (100 μL). The reaction was triggered by adding 100 μL of 300 mM ascorbate and incubated for 1 h at 37°C. An aliquot (0.5 mL) of the reaction mixture was added to 1 mL of TCA (2.8%; w/v; aqueous solution), then 1 mL of 1% aqueous TBA were added to the reaction mixture. The mixture was heated for 15 min on a boiling water bath, cooled and the absorbance of methanol extracts and their various fractions at 50–250 µg/mL for hydroxyl radical scavenging activity was measured at 532 nm against a blank (the same solution but without leaf extract extract).

The scavenging activity on hydroxyl radical was calculated as follows:

$$\text{Scavenging}\;\text{activity}\;\left(\text{\%}\right)=\left[\frac{\text{A}0-\text{A}1}{\text{A}0}\right]\times 100$$ (3)

EC50, which is the concentration (μg/mL) of sample required for 50% scavenging of OH^•^, was calculated from the graph plotted for scavenging percentage against the sample concentration. Gallic acid and ascorbic acid were used as positive controls in these assays. All samples were analyzed in triplicate.

#### 2.7.4. Superoxide anion radicle scavenging assay

Superoxide anion radical scavenging activity was measured via the riboflavin-light-NBT system [47]. Leaf extract sample (1 mL) was mixed with 0.5 mL of phosphate buffer (50 mM, pH 7.6), 0.3 mL riboflavin (50 mM), 0.25 mL PMS (20 mM) and 0.1 mL NBT (0.5 mM). The reaction was triggered by illuminating the reaction mixture using a fluorescent lamp. After incubation for 20 min, the absorbance was measured at 560 nm. Ascorbic acid was used as a standard. The scavenging ability of the plant methanol extracts and their different fractions at 50–250 µg/mL was determined using the following equation:

$$\text{Scavenging}\;\text{effect}\;\left(\text{\%}\right)=\left[\frac{\text{A}0-\text{A}1}{\text{A}0}\right]\times 100$$ (4)

EC50, which is the concentration (μg/mL) of sample required for 50% scavenging of superoxide radical anion, was calculated from the graph plotted for scavenging percentage against the sample concentration. Gallic acid and ascorbic acid were used as positive controls in these assays. All samples were analyzed in triplicate.

#### 2.7.5. Reducing power assay

Estimation of reducing power was based on reduction of Fe (III) to Fe (II) in the presence of the solvent fractions [48]. The formation of Fe (II) was monitored by measuring the change in absorbance of Perl’s Prussian blue at 700 nm. Various concentrations of the sample (2 mL) were mixed with 2 mL of phosphate buffer (0.2 M, pH 6.6) and 2 mL of potassium ferricyanide (10 mg/mL). The mixture was incubated at 50°C for 20 min followed by addition of 2 mL of trichloroacetic acid (100 mg/L). The mixture was centrifuged at 3000 rpm for 10 min to collect the upper layer of the solution. An aliquot (2 mL) from each of the mixtures was mixed with 2 mL of distilled water and 0.4 mL of 0.1% (w/v) fresh ferric chloride. After 10 min, the absorbance of methanol extracts and their different fractions at 50–250 µg/mL was measured at 700 nm. A higher absorbance of the reaction mixture indicated a greater reducing power.

### 2.8. Lipoxygenase (LOX) inhibition assay

Lipoxygenase inhibition activity was assayed following the method described by Wu [49] with slight modifications. A mixture of 1 mL of sodium borate buffer (0.1 M, pH 8.8) and 10 μL of soybean LOX (8,000 U/mL) was incubated with 10 μL of plant extract sample at room temperature for 10 min. The reaction was started by the addition of 10 μL of linoleic acid substrate (10 mM). The absorbance at 234 nm of the resulting mixture was measured each min (for 6 min). Inhibition of LOX was calculated using the following equation:

$$\text{Inhibition}\;\left(\text{\%}\right)=\left[\frac{\text{absorbance}\;\text{of}\;\text{the}\;\text{control}-\text{absorbanceof}\;\text{the}\;\text{sample}}{\text{absorbance}\;\text{of}\;\text{the}\;\text{control}}\right]\times 100$$ (5)

The effective concentration (μg/mL) at which LOX activity is inhibited by 50% (IC_50_) was determined graphically. Nordihydroguaiaretic acid (NDGA) was used as a positive control.

### 2.9. Antimicrobial activities

#### 2.9.1. Pathogenic bacterial and fungal strains used

*Bacteria*. *Staphylococcus aureus*, ATCC 6538; *Pseudomonas aeruginosa*, ATCC 7221; *Klebsiella pneumonia*, MTCC 618 and *Bacillus subtilis*, ATCC 6059 were obtained from the Department of Microbiology, Quaid-i-Azam University, Islamabad, Pakistan.

*Fungi*. *Aspergillus fumigatus*, FCBP-MF-923; *Aspergillus flavus*, FCBP-PTF-1265 and *Fusarium oxysporum*, FCBP-MF-1152 were obtained from the First Fungal Culture Bank of Pakistan (FCBP), University of the Punjab, Pakistan.

The bacterial isolates were first sub-cultured in a nutrient broth (Sigma) and incubated at 37°C for 18 h. The fungal isolates were sub-cultured on SDA (Merck) for 7 d at 25°C.

*Positive and negative control*. Streptomycin (30 µg/mL) and terbinafine (1 mg/mL) were used as positive controls for antibacterial and antifungal assays, respectively. Dimethyl sulfoxide (DMSO) was used as negative control for the antibacterial and antifungal assays.

#### 2.9.2. Antibacterial activity

Antibacterial activity of the methanol, chloroform, ethyl acetate and aqueous extracts of *J*. *mimosifolia* was determined via the agar-well diffusion method [50]. Petri plates were prepared by pouring 75 mL of MH agar and allowing it to solidify. Freshly prepared bacterial inoculum was spread evenly using a sterile cotton swab on the entire agar surface. A hole was then made with a sterile cork borer (6 mm) and 100 µL of each extract was poured into the wells. The Petri plates were then allowed to cool at room temperature for 1 h and incubated at 37°C for overnight. Controls were run in parallel whereby the solvent was used to fill the well. The plates were observed for zones of inhibition after 24 h and results were compared with those of the positive control containing streptomycin (30 µg/mL).

#### 2.9.3. Determination of Minimum Inhibitory Concentration (MIC)

The determination of MIC of all the extracts of *J*. *mimosifolia* was carried out via a microdilution method [51] using nutrient broth. Plant extracts were dissolved in 10% DMSO and two-fold dilutions prepared with culture broth. Each test sample and growth control (containing broth and DMSO, without plant extract/antimicrobial compound was inoculated with 10 µL of bacterial suspension containing 5×10^6^ CFU/mL. A 10-μL solution of resazurin (270 mg resazurin tablet dissolved in 40 mL of sterile water) was also added to each sample and incubated for 24 h at 37°C. Bacterial growth was detected by reading the absorbance at 500 nm and indicated by a color change from purple to pink or colorless (assessed visually). MIC was defined as the lowest leaf extract concentration at which the color changed or the highest dilution that completely inhibited the bacterial growth. Experiments were carried out in triplicate to test each dilution for each bacterial strain to determine MIC values.

#### 2.9.4. Assay for antifungal activity

The agar tube dilution method was used to determine the antifungal activity of the leaf extracts [52]. Samples were prepared by dissolving plant extracts in DMSO. Culture media was prepared by dissolving 6.5 g of potato dextrose agar per 100 mL distilled water (pH 5.6). SDA (10 mL) was dispensed in screw-capped tubes and autoclaved (121°C for 21 min). Tubes were allowed to cool at 50°C and the SDA was loaded with 67 μL of extract pipetted from the stock solutions. The tubes containing the media were then allowed to solidify in slanting position at room temperature. The tubes containing solidified media and plant extract were inoculated with a 4-mm-diameter piece of inoculum taken from a 7 d-old culture of fungus. Controls were run in parallel whereby the respective solvent was used instead of plant extract. The test tubes were incubated at 28°C for 7 d. Cultures were examined twice weekly during the incubation. Readings were taken by measuring the linear length (mm) of fungus in the slant, and growth inhibition was calculated with reference to negative control.

Percentage inhibition of fungal growth for each concentration of compound was determined as follows:

$$\text{Fugal}\;\text{growth}\;\text{inhibition}\;\left(\text{\%}\right)=\left[1-\frac{\text{Linear}\;\text{growth}\;\text{in}\;\text{test}\left(\text{mm}\right)}{\text{Linear}\;\text{growth}\;\text{in}\;\text{control}\left(\text{mm}\right)}\right]\times 100$$ (6)

### 2.10. Cytotoxicity assay

The standard protocol for the assessment of cellular toxicity measures the ability of cultured cells to proliferate in the presence of a test sample, and subsequently quantitates total protein content with sulforhodamine B (SRB) dye as a measure of the percentage of surviving cells. The cytotoxic potential of methanol extracts and their different fractions was determined in the human lung carcinoma (LU-1) and human prostrate carcinoma (LnCaP) cell lines at 1.562–50 µg/mL in DMSO. Cultures (3 x 10^4^ cells/mL) were seeded in 96-well plates, and six two-fold serial dilutions of test samples in 10% DMSO (10 μL) were added to each well. The plates were incubated in air humidified and containing 5% CO_2_, at 37°C in incubator (Biotech) for 72 h, after which cell viability was determined with SRB staining following the standard protocol [53]. EC_50_ values were determined as the concentration of sample required to inhibit cell growth by 50% relative to a control treated with 0.5% DMSO. Colchicine (Sigma-Aldrich; purity > 96 by HPLC) was used as the positive control compound.

### 2.11. GC-MS analysis of phytochemical compounds

GC-MS analysis of crude organic extracts was performed on a PerkinElmer Clarus 600 GC System fitted with a Rtx-5MS capillary column (30 m x 0.25 mm internal diameter, 0.25 μm film thickness; maximum temperature, 350°C), coupled to a Perkin Elmer Clarus 600C MS. Ultra-high purity helium (99.99%) was used as carrier gas at a constant flow rate of 1.0 mL/min. The injection, transfer line and ion source temperatures were all 290°C. The ionizing energy was 70 eV. Electron multiplier voltage was obtained from autotune. The oven temperature was programmed from 60°C (hold for 2 min) to 280°C at a rate of 3°C/min.

The crude samples were diluted with appropriate solvent (1/100, v/v) and filtered. The particle-free diluted crude extracts (1 μL) were injected using a syringe into the injector with a split ratio 30:1. All data were obtained by collecting the full-scan mass spectra within the scan range 40–550 amu. The percentage composition of the crude extract constituents was expressed as a percentage by peak area. The identification and characterization of chemical compounds in various crude extracts was based on GC retention time. The mass spectra were computer matched with those of standards available in mass spectrum libraries.

Interpretation on Mass-Spectrum GC-MC was conducted using the database of National Institute Standard and Technology (NIST). The spectrum of the unknown components was compared with the spectrum of known components stored in the NIST library as well as by comparison of the retention time. The name, molecular weight and structure of the components of the test material was ascertained.

### 2.12. Statistical analysis

Three biological replicate extracts were analyzed for each assay described above. Results were expressed as the mean ± standard deviation of mean (SD). The data generated from quantitative assays for phytochemicals were subjected to ANOVA using Statistix version 8.1. Comparison among mean values was made by Least Significant Difference (LSD) to test significant differences at P< 0.05 [54]. Linear regression analysis was used to calculate IC_50_ values. Linear correlations were analyzed by using regression in R software (3.2.2.).

## 3. Results and discussion

### 3.1. Determination of phytochemicals and identification of polyphenolic compounds

Plant extracts are complex mixtures of chemical compounds known to play important role in several biological activities. In this study the qualitativeq analysis of phytochemicals of *Jacaranda mimosifolia* revealed the presence of alkaloids, phenolics, flavonoids tannin and saponin in all the leaf extract fractions. Terpenoids were detected in all the fractions except EAF. Phlobatannins (tannins that with hot dilute acids yield a phlobaphene) were observed only in ME and EAF, whereas coumarin was found only in ME and CF (Table 1). Previously, qualitative phytochemical analysis of *J*. *mimosifolia* leaf extract showed the presence of alkaloids, phenolics, flavonoids, tannins and saponins [55]. Flower extracts of *J*. *mimosifolia* were found to be rich in phenolics, flavonoids, terpenoids and quinones [56].

**Table 1:** Presence/absence of major phytochemical groups in *J*. *mimosifolia* leaf extracts and fractions.

Class of metabolites	ME	AE	CF	EAF
Alkaloids	+	+	+	+
Phenolics	+	+	+	+
Flavonoids	+	+	+	+
Tannins	+	+	+	+
Saponins	+	+	+	+
Terpenoids	+	+	+	−
Phlobetannins	+	−	−	+
Coumarins	+	−	+	−

ME, methanol extract; AE, aqueous extract (from ME residue); CF, chloroform fraction from ME crude extract; EAF, ethyl acetate fraction from ME.

For natural product extracts,products the amounts of total phenolic and flavonoid compounds are considered as significant parameters when evaluating the quality of the extract as well as its biological potential [57]. In this study ME had the maximum phenolic, flavonoid, flavonol, tannin and ornithodiphenol contents (125, 85, 19, 2.6 and 119 mg/g, respectively), followed by CF (Table 2). Crude extracts of herbs, cereals, fruits, vegetables and other plant materials rich in phenolics are being used increasingly in the food industry because they retard oxidative degradation of lipids, thereby improving the quality and nutritional value of food [58]. The methanol extract had the highest TPC, with the residual aqueous fraction containing much lower levels of phenolics, which is in agreement with other reports [59, 60]. Phenolic compounds are known to donate hydrogen to reduce oxidative damage by detoxifying free radicals [61, 62]. These compounds are also reported to activate the endogenous system of antioxidants and repressed lipid peroxidation of erythrocytes in humans [63, 64].

**Table 2:** Total phenolic, flavonoid, flavonol, tannin and *ortho*-diphenol contents in *J*. *mimosifolia* leaf extracts and fractions.

Extract/ fraction	TPC (mg GAE /g DW)	TFC (mg RE/g DW)	TFLC (mg RE/g DW)	TT (mg/g DW)	ORTH (mg GAE/g DW)	Extract yield (%)
**ME**	125.8^a^±3.3	85.7^a^±0.32	19.1^a^±0.14	2.6^a^±0.16	119.4^a^±2.7	16.3
**AE**	54.6^c^±2.8	53.1^c^±0.27	9.28^d^±0.16	0.77^c^±0.21	48.3^d^±1.8	12.2
**CF**	93.4^b^±3.2	76.9^b^±0.35	14.6^b^±0.11	1.98^b^±0.18	84.6^b^±2.3	9.6
**EAF**	92.7^b^±2.5	75.3^b^±0.29	12.4^c^±0.09	1.96^b^±0.16	82.9^c^±2.2	8.5

TPC, total phenolic content; TFC, total flavonoid content; TFLC, total flavonol content; TT, total tannin; ORTH, *Ortho*-diphenols

Data represent mean ± standard deviation (SD) of three biological replicates. All mean values in the same column with different letters are significantly different from each other at P< 0.05.

### 3.2. HPLC analysis of main polyphenolic compounds

HPLC analysis revealed the presence of various polyphenolic compounds in *J*. *mimosifolia* leaf extract fractions identified against eight standards; i.e., apigenin, caffeic acid, catechin, gallic acid, kaempferol, isoquercetin, rutin and *trans*-cinnamic acid (Table 3). All of the eight compounds were detected in ME, with isoquercetin, rutin, catechin and caffeic acid in the highest concentration (14.91, 11.55, 9.68 and 7.34 mg/g extract, respectively) compared to other fractions. In AE, only caffeic acid, gallic acid and catechin were detected. With the exception of apigenin, all of the previously mentioned polyphenolic compounds were detected in the CF, with isoquercetin, catechin, rutin and caffeic acid as the major components. Finally, the EAF was found to contain low concentrations of caffeic acid, catechin, gallic acid, rutin and *trans*-cinnamic acid compared to ME and CF.

**Table 3:** Major polyphenolic compounds of *J*. *mimosifolia* leaf extracts and fractions as quantified by HPLC analysis.

Compound identified	ME		AE		CE		EAE	
	RT (min)	Contents (mg/g extract)	RT (min)	Content (mg/g extract)	RT (min)	Content (mg/g extract)	RT (min)	Contents (mg/g extract)
apigenin	4.5	3.31^e^±0.93	-	nd	-	-	-	nd
caffeic acid	21.4	7.34^cd^±1.5	27.7	6.31^d^±1.6	22.3	7.16^cd^±1.3	21.9	6.68^d^±1.1
catechin	26.7	9.68^bc^±1.7	27.3	6.75^d^±1.1	26.9	9.62^bc^±1.5	26.7	7.93^cd^±1.3
gallic acid	11.6	0.97^g^±0.05	13.7	0.39^i^±0.06	11.8	0.88^gh^±0.07	12.4	0.64^h^±0.09
kaempferol	3.3	2.24^f^±0.92	-	nd	4.3	2.18^f^±1.05	-	
isoquercetin	5.8	14.91^a^±2.3	-	nd	6.4	12.76^ab^±1.7	-	
rutin	8.5	11.55^b^±1.9	-	nd	8.9	9.5^bc^±1.5	9.2	8.81^c^±1.4
*trans*-cinnamic acid	7.3	2.97^ef^±0.85	-	nd	7.9	2.67^ef^±0.67	8.5	2.19^f^±0.75

Data represent mean ± standard deviation (SD) of three biological replicates. All mean values in the same column with different letters are significantly different from each other at P< 0.05.

The methanol leaf extract fraction of *J*. *mimosifolia* was found to contain some important flavonoids; i.e., apigenin, caffeic acid, catechin, gallic acid, kaempferol, isoquercetin, rutin and *trans*-cinnamic acid. The maximum total polyphenolic and flavonoid content, as well as antioxidant activity, has been reported in leaf extracts of *Amaranth hypochondriacus*. They also mentioned the highest polyphenolic, flavonoid content and antioxidant potential in leaf extracts compared to different other parts of plants [65, 66]. Flavonoids among all the phenolic compounds are known as the most important due to their strong antioxidant potential [67]. Flavonoids have potent antimicrobial and antioxidant potential and are thought to have positive effects on human health [68]. It was reported that the flavonoid, rutin, also present in *J*. *mimosifolia* extract, promoted the lipid peroxidation inhibition in heart tissues of rats exposed to oxidative stress [69]. Henneberg et al. [70] also reported the efficacy of rutin to detoxify oxidative species in human erythrocytes under oxidative stress.

Studies on flavonoid derivatives have shown a wide range of antibacterial, anti-allergic, antiviral, anti inflammatory [71] and anticancer activities [72]. Therefore, the presence of flavonoids in *J*. *mimosifolia* supports their folkloric use against various skin infections.

### 3.3. Determination of antioxidant activities

**Fig 1:**
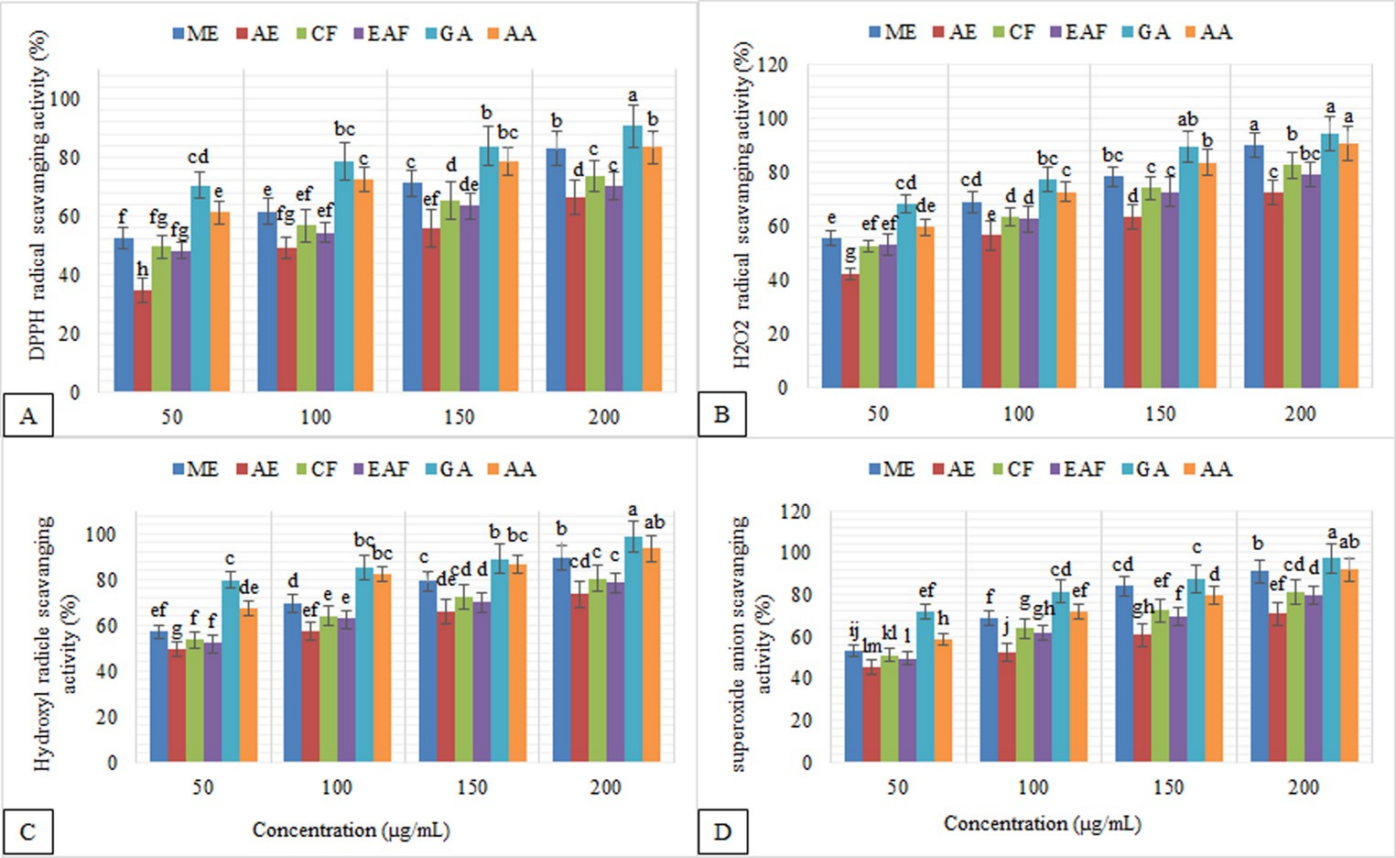
Antioxidant activities of *J*. *mimosifolia* leaf extracts and fractions at various concentrations. Data represent mean ± standard deviation (SD) of three biological replicates. **(A)** DPPH radical scavenging activity, **(B)** Hydrogen peroxide radical scavenging activity, **(C)** Hydroxyl radical scavenging activity, **(D)** Superoxide radical scavenging activity. GA, gallic acid; AA, ascorbic acid.

The scavenging power of *J*. *mimosifolia* fractions on DPPH, hydroxyl radical, hydrogen peroxide and superoxide radical were in the following order: ME > CF > EAF > AE (Fig 1). The EC_50_ values for scavenging DPPH radicals for the ME and CF were 48±1.3 and 52±2.5 μg/mL, respectively (Table 4). In agreement with these results, Chen et al. [73] found that, for *Jacaranda acutifolia* flower, the DPPH radical scavenging ability of ME was relatively high compared to the valuefor the ethyl acetate fraction (EC_50_ = 0.049 mg/mL). Naz and Bano [74] also reported that methanol extracts of *Lantana camara* exhibited the maximum DPPH scavenging ability.

**Table 4:** Radical scavenging activities of *J*. *mimosifolia* leaf extracts and fractions.

Extract/standard compound	EC_50_ values (μg/mL) for radical scavenging			
	DPPH	H_2_O_2_	^•^OH	O_2^-^_
ME	48^c^±1.3	45^bc^±2.4	42^bc^±1.3	46^c^±1.3
AE	120^a^±3.4	93^a^±3.5	85^a^±3.6	93^a^±3.5
CF	52^bc^±2.5	48^b^±1.3	43^b^±2.4	48^c^±2.4
EAF	55^b^±3.1	47^b^±2.7	45^b^±2.7	55^b^±2.7
GA	35^de^±1.1	38^cd^±2.5	28^d^±1.1	35^d^±1.3
AA	40^d^±2.6	42^c^±2.8	39^c^±1.6	42^cd^±1.4

Data represent mean ± standard deviation (SD) of three biological replicates. GA, gallic acid; AA, ascorbic acid

ROS, including hydroxyl radical, superoxide radical, hydrogen peroxide and peroxynitryl, are continuously being synthesized in living systems because of metabolic reactions [29] and they are known to cause several diseases associated with oxidative stress. Therefore such ROS need to be detoxified/scavenged with the help of natural products to enhance the host defense system. The scavenging effects of different fractions of *J*. *mimosifolia* on hydrogen peroxide, hydroxyl radical and superoxide radical were concentration-dependent (50–200 μg/mL; Fig 1). ME exhibited a higher hydrogen peroxide scavenging activity (EC_50_ 45±2.4 μg/mL; Table 4) compared to that of the standards, gallic acid and ascorbic acid (38±2.5 and 42±2.8 μg /mL, respectively). CF and EAF exhibited a moderate hydrogen peroxide scavenging activity (EC_50_ 48±1.3 and 47±2.7 μg/mL, respectively). EC_50_ values of the fractions in scavenging hydrogen peroxide were significantly different (P < 0.05) from the EC_50_ values obtained for gallic acid and ascorbic acid.

The EC_50_ value for hydroxyl radical scavenging activity for the ME and CF was 42±1.3 and 43±2.4 μg/mL, while for EAF it was 45±2.7 μg/mL (Table 4). The strong antioxidant activity of ME and CF (Fig 1) showed that these fractions can be the excellent source of natural antioxidants.

The hydroxyl radical and its associated radicals are known as very detrimental ROS, because in various biomolecules they are mostly responsible for oxidative injury [75]. Superoxide anion is another major biological source of ROS [75, 76]. Although the superoxide radical is a weak oxidant, it gives rise to the generation of powerful and dangerous hydroxyl radicals as well as singlet oxygen, both of which contribute to oxidative stress [77]. When compared to gallic acid and ascorbic acid, the superoxide scavenging activity of EAF was found to be low (P < 0.05) (Fig 1). However, ME and CF (EC_50_ 46±1.3 and 48±2.4 μg/mL, respectively) behaved as powerful superoxide anion scavengers. Though the antioxidant potential of the fractions was found to be lower (at P < 0.05) than those of the standards, ME and CF had substantial antioxidant activity. Most of the phenolic content of the *J*. *mimosifolia* leaves was found in these two fractions and thus these compounds could be responsible for the observed high antiradical properties of these fractions. These results are in accordance with Saeed et al [78] who suggestedsuggest that phytochemical constituents of plant extracts have the ability to scavange the possible damage by donating hydrogen to a free radical.

**Fig 2:**
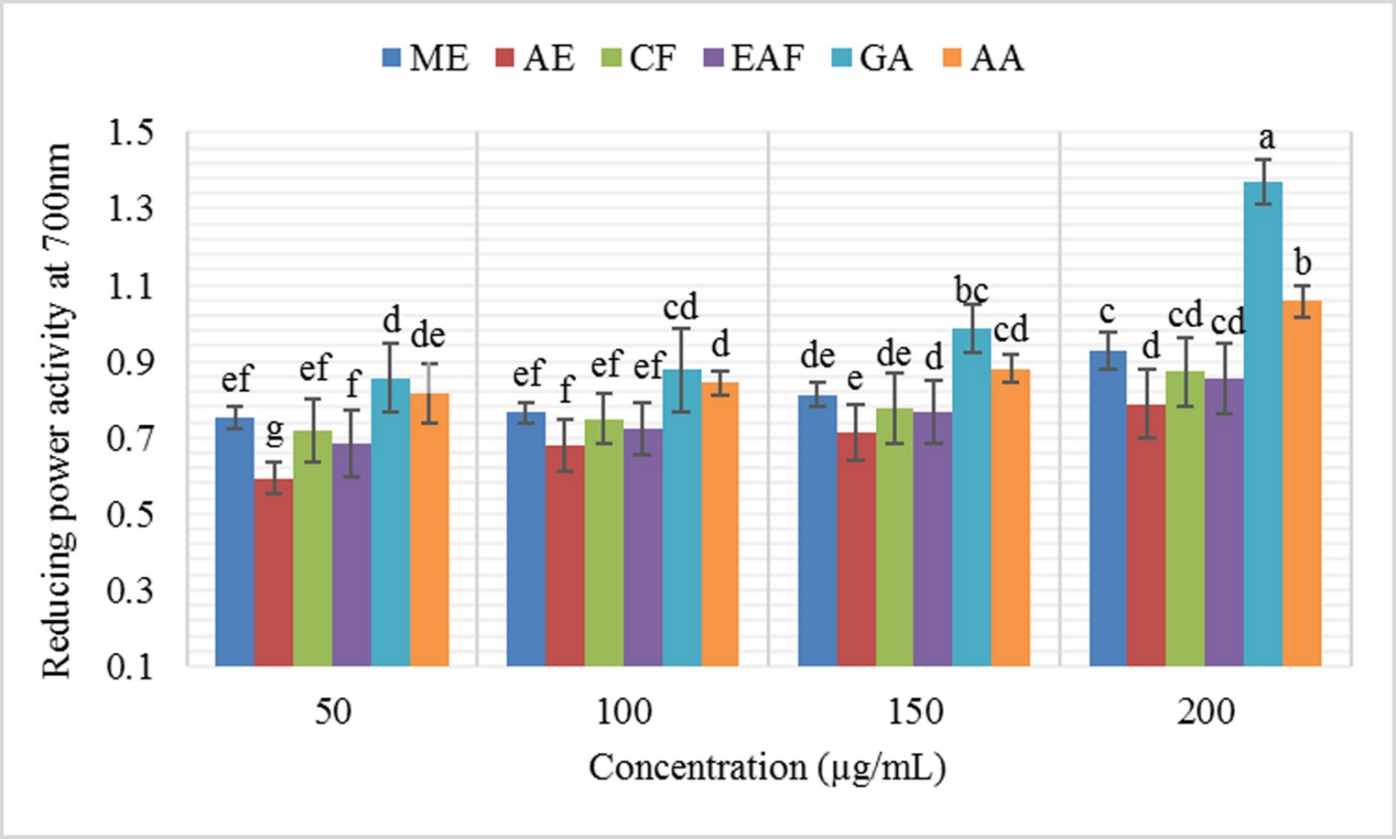
Reducing power of *J*. *mimosifolia* leaf extracts and fractions at different concentrations. Data represent mean ± standard deviation (SD) of three biological replicates.

The reducing power of all leaf extract fractions (50–200 μg/mL) from *J*. *mimosifolia* was found to increase with concentration of the sample (Fig 2). The ranking order for reducing power was ME > CF > EAF > AE. Significantly higher reducing power (0.929±0.09 at 200 μg/mL) was found in the ME fraction. All extracts and fractions exhibited reducing power (Fig 2), which is in accordance with several results in the literature [79, 80, 81]. Methanol extract showed the maximum reducing power, which is in agreement with Naz and Bano [74] who reported the highest reducing power ability of methanol extracts of *Lantana camara*.

Eugenol extracts exhibited significant antioxidant potential as measured using DPPH radical scavenging (81%) and reducing power (1.12) at 1.0 μM/ml and 0.1 μM/ml, respectively [82]. Hossain and Shah [83] reported higher flavonoids content and antioxidant activity as well as positive correlation between them, in chloroform extract of *Merremia borneensis*. Superoxide anion, hydroxyl radical and hydrogen peroxide radical scavenging activities were reported higher in *Torilis leptophylla* chloroform extract compared to its ethyl acetate extract [78].

The methanol extract of *J*. *mimosifolia* exhibited relatively comparable antioxidant properties to the commonly used antioxidant ascorbic acid. Differences in antioxidant activities could be related to the antioxidant components extracted from the different *J*. *mimosifolia* fractions. Several significant correlation coefficients were found between phenolic compounds and DPPH (R^2^ = 0.942*), H_2_O_2_ (R^2^ = 0.979**), OH (R^2^ = 0.964*), O_2_^-1^ (R^2^ = 0.995**) and reducing power (R^2^ = 0.992**) (Table 5). Flavonoids also showed significant correlations with hydrogen peroxide (R^2^ = 0.939**), O_2_^-1^ (R^2^ = 0.954*) and reducing power (R^2^ = 0.967*). These correlations strongly suggest the involvement of phenolic compounds in the antioxidant activities. This result is in agreement with previous reports that have demonstrated a linear correlation between TPC and the reducing antioxidant capacity of plant extracts [84, 85].

**Table 5:** Correlation matrix between the quantified phytochemicals and antioxidant activities.

	TPC	TFC	TFLC	TT	ORTH	DPPH	H_2_O_2_	^•^OH	O_2^-^_
TFC	0.97*								
**TFLC**	0.97*	0.92							
**TT**	0.99**	0.997***	0.93						
**ORTH**	0.998***	0.96*	0.976*	0.976*					
**DPPH**	0.942*	0.859	0.99**	0.883	0.959*				
**H**_**2**_**O**_**2**_	0.979**	0.939*	0.998***	0.951*	0.985**	0.981**			
^**•**^**OH**	0.964*	0.887	0.989**	0.911	0.978*	0.996**	0.985**		
**O**_**2**^**-**^_	0.995**	0.954*	0.986**	0.969*	0.999*	0.970**	0.993**	0.985**	
**RP**	0.992**	0.967*	0.987**	0.976*	0.993**	0.961*	0.995**	0.973*	0.996**

Significance of correlations: *p < 0.05: Significant correlation;

** p < 0.01: Very significant correlation;

***p < 0.001: Extremely significant correlation

### 3.4. Lipoxygenase (LOX) inhibition assay

**Fig 3:**
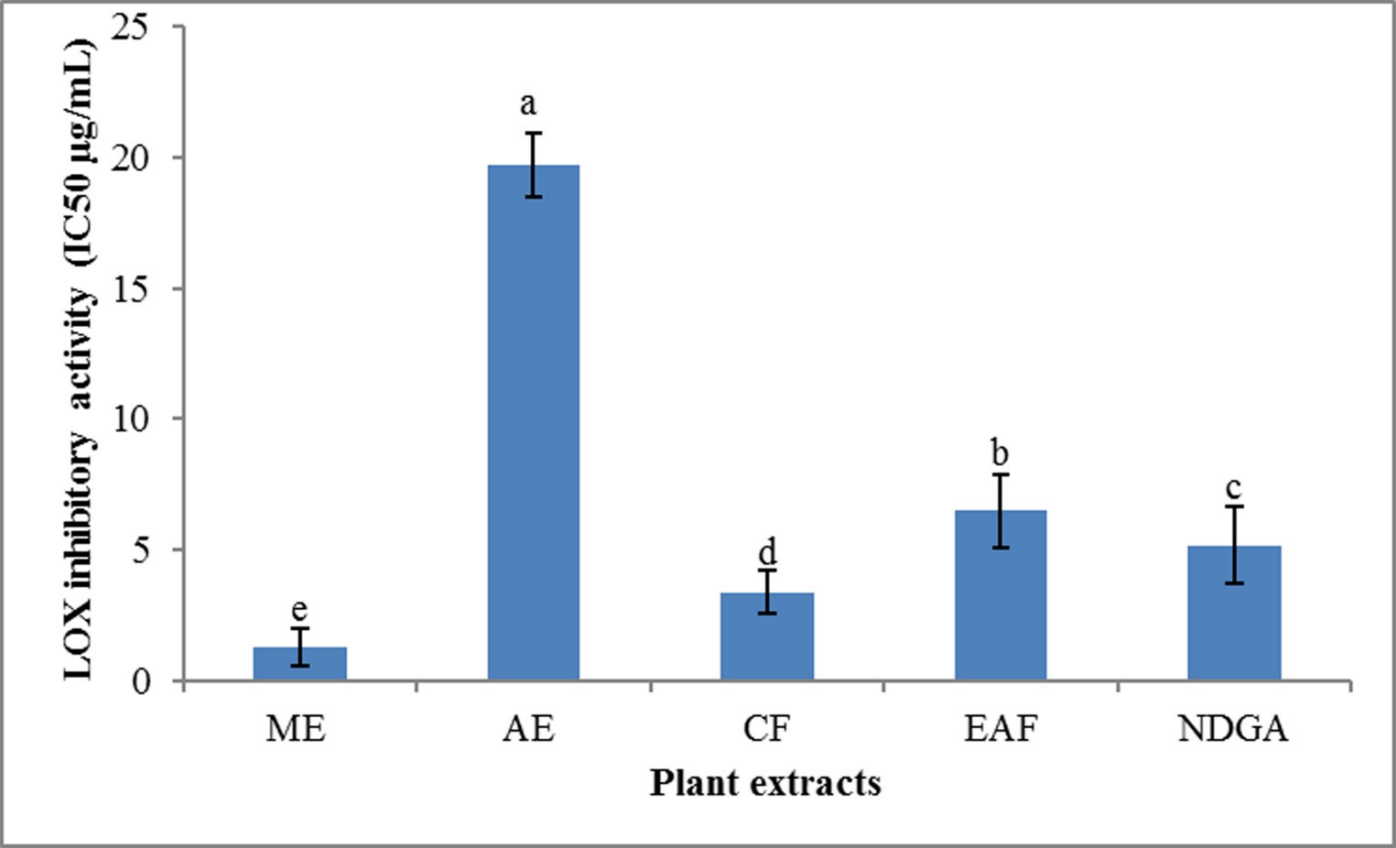
LOX inhibitory activities of *J*. *mimosifolia* methanol extract and soluble fractions expressed as IC_50_ (μg/mL). Data represent mean ± standard deviation (SD) of three biological replicates.

ME, CF and EAF showed a substantial ability to inhibit LOX activity (IC_50_ = 1.3, 3.4 and 6.5 μg/mL, respectively), while AE showed much lower activities (IC_50_ = 19.7 μg/mL) (Fig 3).

Both the ME and CF exhibited significantly higher LOX inhibitory activity than that of an NDGA positive standard (IC_50_ = 5.2 μg/mL). Lipoxygenase is involved in arachidonic acid metabolism, generating various biologically active leukotrienes that play an important role in inflammation. Lipoxygenases are the key enzymes in the biosynthesis of leukotrienes which play an important role in the incidence of several inflammatory diseases such as cancer, arthritis, asthma, allergic diseases [86], chronic pain [25] and inflammatory bowel disease [28]. Therefore, lipoxygenases are potential targets for rational drug design and discovery of mechanism-based inhibitors for the treatment of a variety of disorders such as bronchial asthma, inflammation, cancer and autoimmune diseases [87].

Oxidative substances (ROS) are important in causing inflammation; hence a plant extract with strong antioxidant potential could be a good anti-inflammatory. *Centaurea salicifolia* M.Bieb. ex Willd. chloroform extracts showed strong antioxidant ability, maximum phenolic content, remarkable anticancer and anti-inflammatory activity [88]. These results suggest that *J*. *mimosifolia* has a potentially high anti-inflammatory effect, which might be related to the polyphenolic content and antioxidant activities of the extract. Torres et al., [29] also reported that plants belonging to Bignoniacea family showed good anti-inflammatory activity, which might be because of phytochemical constituents that were found effective against LOX enzyme. Therefore, lipoxygenase inhibitors may be of many medicinal benefits in prevention of these inflammatory cases [86]. A plant with good anti-lipoxygenase or anti-inflammatory potential could be a good candidate for antiallegic, anti-arthritic and anticancer drug.

### 3.5. Antibacterial activity

**Fig 4:**
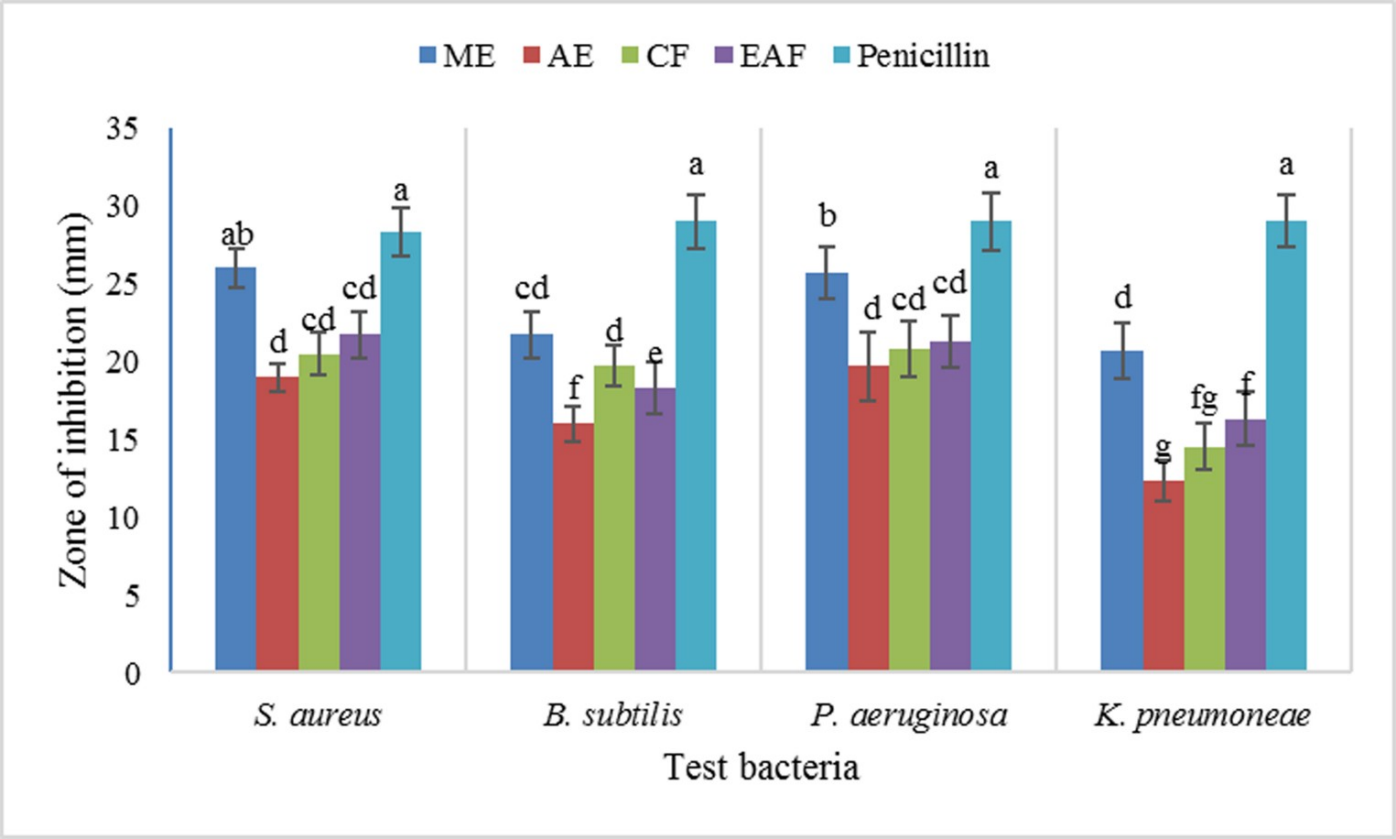
Zone of inhibition of *J*. *mimosifolia* methanol extract and different fractions against pathogenic bacterial strains. Data represent mean ± standard deviation (SD) of three biological replicates.

The results of the antibacterial and antifungal activity varied with the leaf extract/fraction studied. The *in vitro* antibacterial activity of various fractions of *J*. *mimosifolia* was evaluated against two gram-positive (*B*. *subtilis*, *S*. *aureus*) and two gram-negative (*P*. *aeruginosa*, *K*. *pneumoniae*) bacteria. ME showed the maximum antibacterial activity with a zone of inhibition 26 mm and 25.7 mm against *S*. *aureus* and *P*. *aeruginosa*, respectively. ME also exhibited the maximum antibacterial potential against four bacterial isolates (*S*. *aureus*, *B*. *subtilis*, *P*. *aeruginosa* and *K*. *pneumoneae*), followed by EAF > CF > AE (Fig 4).

The MIC values for antibacterial activity of various plant extract fractions obtained against all tested bacteria ranged from 0.01 to 10 mg/mL (Table 6). Gram-positive bacteria such as *S*. *aureus* were inhibited with an MIC value of 1.5 and 2.5 mg/mL for ME and CF, respectively, 10 mg/mL for AE and 5 mg/mL for EAF. In contrast, *B*. *subtilis* was inhibited by ME and CF (both 5 mg/mL) and EAF (10 mg/mL), while the aqueous fraction did not inhibit the growth of this bacterial species. In the case of the gram-negative bacterium, *P*. *aeruginosa*, growth was inhibited by CF (1.5 mg/mL); ME (2.5 mg/mL) and AE (10 mg/mL). *K*. *pneumoniae’s* growth was inhibited by ME (5 mg/mL) and EAF (10 mg/mL), while the other fractions did not inhibit the growth of this bacterial species. From the results we conclude that *S*. *aureus* was the most susceptible while *B*. *subtilis* and *P*. *aeruginosa* were less susceptible. Moderate to high antimicrobial activities of *J*. *cuspidifolia*, *J*. *glabra* and *J*. *mimosifolia* extracts have been reported [89, 90, 91]. The presence of different bioactive constituents, namely saponins, phenolics and flavonoids, in the *J*. *mimosifolia* leaf extract and various fractions might contribute towards the diversity of antibacterial activities. The presence of several phytochemical compounds in *J*. *mimosifolia* can be associated with its different biological activities particularly because of phenolic compounds that are associated with antimicrobial, antioxidant, and anti-inflammatory activities [92, 93]. Several biological activities are ascribed to phenolic and flavonoids including antimicrobial, antiviral, antioxidant, antihepatotoxic, antiulcer, apoptotic and antiproliferative activities [94].

**Table 6:** MIC values for *J*. *mimosifolia* leaf extracts and fractions.

Test Bacteria	MIC (mg/mL)				
	ME	AE	CE	EAE	PC
***Staphylococcus aureus***	1.5^d^±0.02	10.0^a^±0.3	2.5^c^±0.02	5.0^b^±0.1	0.05^g^±0.01
***Bacillus subtilis***	5.0^b^±0.01	Nd	5.0^b^±0.07	10.0^a^±0.05	0.20^f^±0.03
***Pseudomonas aeruginosa***	2.5^c^±0.02	10.0^a^±0.1	1.5^d^±0.03	5.0^b^±0.07	0.05^g^±0.05
***Klebsiella pneumoneae***	5.0^b^±0.1	N.d.	N.d.	10.0^a^±0.2	0.25^e^±0.03

Data represent mean ± standard deviation (SD) of three biological replicates. N.d., not detected

### 3.6. Antifungal activity

Antifungal activity of ME, AE, CF and EAF of *J*. *mimosifolia* was investigated against three fungal strains; i.e., *Aspergillus fumigatus*, *Aspergillus flavus* and *Fusarium oxysporum* (Table 7). ME exhibited good antifungal activity (94%, 91% and 95%) while EAF showed 90%, 88% and 93% inhibition against *A*. *fumigatus*, *A*. *flavus* and *F*. *oxysporum*, respectively. The ranking order of extracts and fractions for antifungal potential was ME > EAF > CF > AE.

**Table 7:** Antifungal potential of *J*. *mimosifolia* extracts and fractions.

Test Fungus	Percentage inhibition (%)				
	ME	AE	CE	EAE	Terbinafine
***Aspergillus fumigatus***	94.2^a^±7.5	76.6^ef^±4.9	82.5^de^±7.7	89.6^c^±5.9	90.9^bc^±5.5
***Aspergillus flavus***	90.6^bc^±6.9	70.9^f^±7.5	79.7^e^±6.5	87.9^cd^±5.5	89.3^c^±6.4
***Fusarium oxysporum***	95.3^a^±6.5	79.3^e^±4.5	85.2^d^±6.5	92.9^b^±6.5	95.7^a^±6.9

Data represent mean ± standard deviation (SD) of three biological replicates.

Secondary metabolites serve as natural sources of a variety of chemical compounds which are known for their antifungal properties [95]. Supporting these results, many medicinal plant extracts are reported for their significant antifungal potential against *A*. *flavus* [60, 74, 96]. The antimicrobial potential of plant extracts could be attributed to the presence of several bioactive compounds, including polyphenols, phenolics, flavonoids and tannins [60, 97]. Baydar et al. 97 confirmed that among all such bioactive compounds, phenolics serve as the most important active compound against fungi as well as bacterial pathogens.

### 3.7. Cytotoxicity

**Fig 5:**
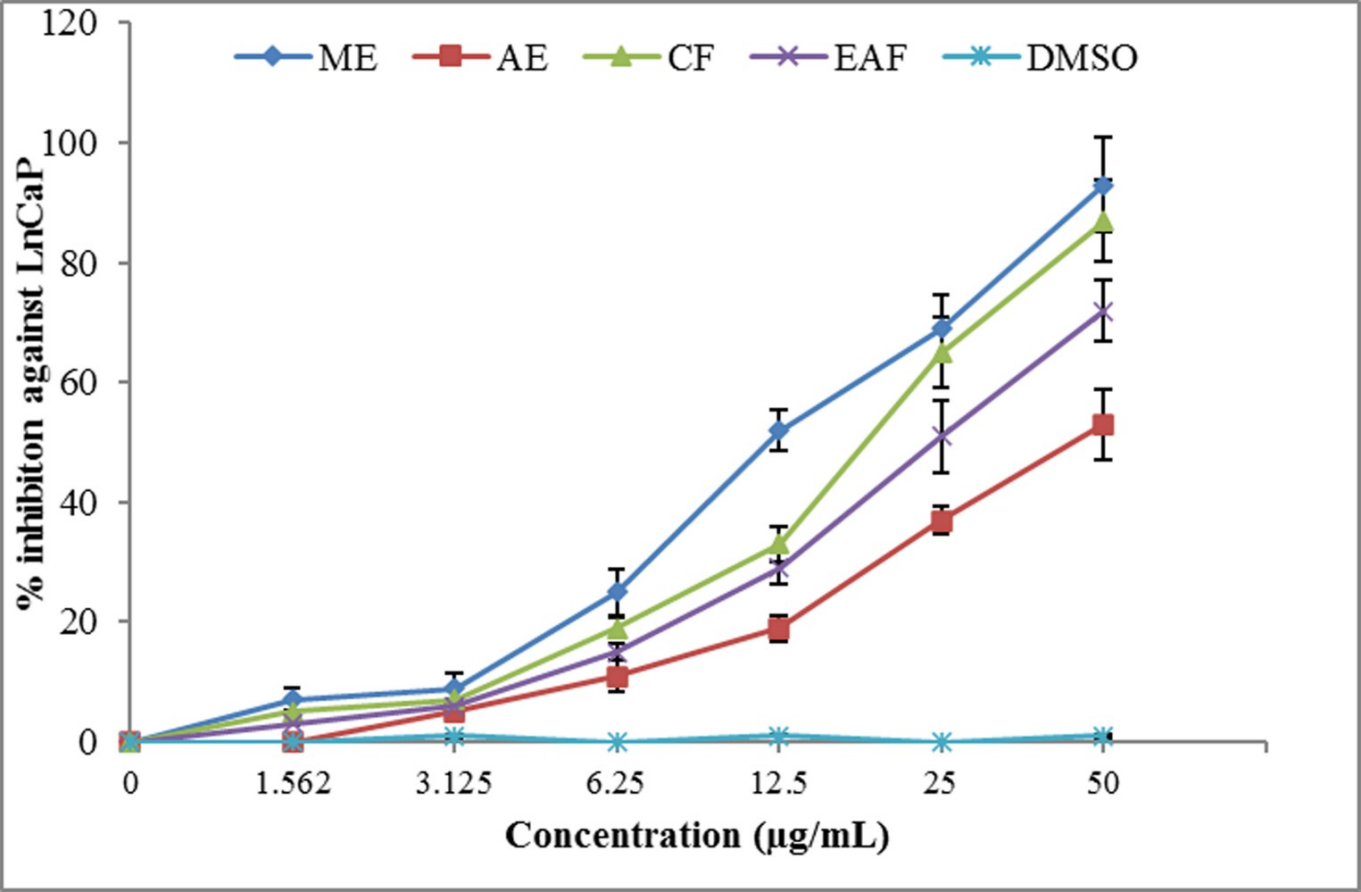
Analysis of cytotoxic activity of *J*. *mimosifolia* extracts and fractions against growth of hormone-dependent prostrate carcinoma (LnCaP-1) cells. Data represent mean ± standard deviation (SD) of three biological replicates.

**Fig 6:**
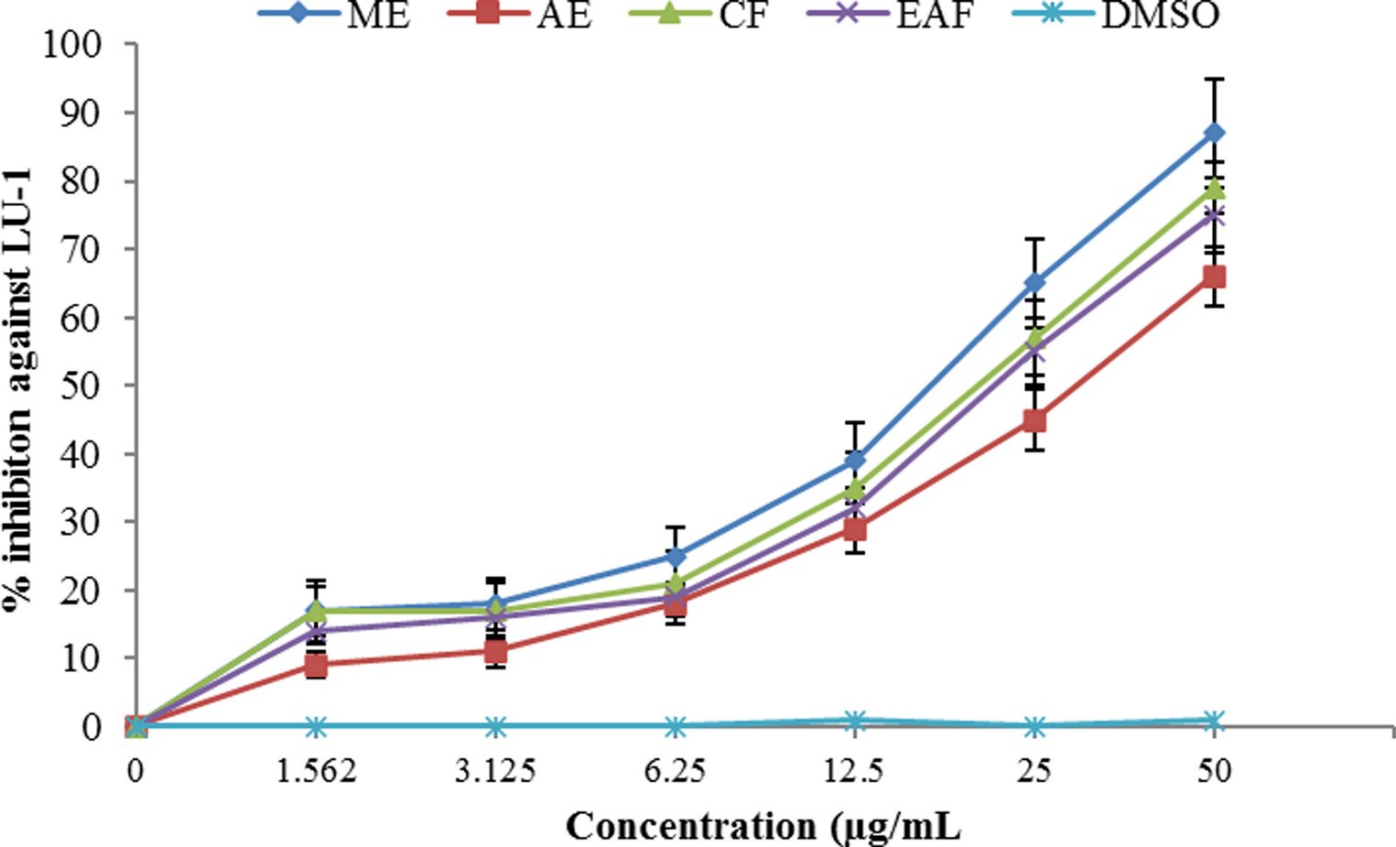
Analysis of cytotoxic activity of *J*. *mimosifolia* extracts and fractions against growth of human lung carcinoma (LU-1) cells. Data represent mean ± standard deviation (SD) of three biological replicates.

The inhibitory effect of cell growth was observed to be concentration dependent (Figs 5 and 6). ME, once again, showed the strongest cytotoxic effect against human prostate carcinoma (LnCaP) and human lung carcinoma (LU-1) cell lines (IC_50_ = 10.7 and 17.3 μg/mL, respectively) followed by CF (IC_50_ = 18.5 and 20 μg/mL, respectively) and EAF (IC_50_ = 23 and 20 μg/mL, respectively), whereas the lowest effect was found for AE (IC_50_ = 50 and 35 μg/mL, respectively). The fact that the ME exhibited the highest cytotoxic activity suggests that antioxidants play a major role in the cytotoxicity. These results are in accordance with Sekerler et al. [88] who reported that *Centaurea salicifolia* M.Bieb. ex Willd. chloroform extracts showed strong antioxidant ability, maximum phenolic content, remarkable anticancer, cytotoxic and anti-inflammatory activity. Therefore it can be supposed that other than antioxidants, high phenolic and flavonoid contents of *J*. *mimosifolia* ME are involved in the cytoxic activity. A study reported a good correlation between the flavonoid contents, antioxidant activities and cytotoxic activity (hepatocarcinoma cell line HepG2) of *C*. *cuneifolia*, *C*. *kilaea*, *C*. *salicifolia* chloroform extracts [88].

Interestingly, *J*. *mimosifolia* exhibited a higher inhibition potential against human prostate carcinoma (LnCaP) and human lung carcinoma (LU-1) cell lines than a previous study of the methanol extract of *Jacaranda obtusifolia* H. B. K. ssp. *rhombifolia* Gentry in which twig extracts exhibited anticancer activity against the NCI-H187 (small cell lung cancer) cell line with an IC_50_ of 23.2 mg/mL [98]. Cinnamaldehyde displayed significant cytotoxic potential (75%) against a prostate cancer (PC-3) cell line [99]. The high anticancer potential of *J*. *mimosifolia* aqueous extract implies that phenolic compounds may not be the only compounds responsible for the cytotoxicity of *J*. *mimosifolia* and suggests the possibility of other compounds with strong activity. This particular point needs to be further investigated. To the best of our knowledge, this study represents the first time *J*. *mimosifolia* ME has been demonstrated to possess anticancer potential against human lung carcinoma and hormone-dependent prostate carcinoma cell lines.

### 3.8 GC-MS analysis

The use of medicinal plants in the treatment of various human ailments depends on their phytochemical constituents. Our investigation of the presence of various active constituents in crude methanol extract and various fractions [chloroform, ethyl acetate and residual aqueous extract] indicated that methanol extracted the greatest range of phytochemicals from the plant. Hence, we used only the methanol extract for the GC–MS study. Gas chromatography-mass spectrometry (GC-MS) of the methanol leaf extract of *Jacaranda mimosifolia* revealed the presence of 15 bioactive compounds with different retention times (Table 8). The following bioactive compounds were found in the methanolic extracts: 2-nonadecanone 2, 4-dinitrophenylhydrazine; ethyl iso-allocholate; 4-(3-hydroxy-1-propenyl)-2-methoxyphenol; hexadecanoic acid (palmitic acid); 1-β-d-ribofuranosyl-3-[5-tetraazolyl]-1,2,4-triazole; 2,4-bis(1,1-dimethylethyl)-phenol; Z,Z-4,16-octadecadien-1-ol acetate; 3-pyridinol, hexadeca-9-en-1-ol (phenolic and alcoholic compounds); 1-methyl-2-(3-methylpentyl)-cyclopropane; hexadecanoic acid, methyl ester, 9,12-octadecadienoic acid(Z,Z) (fatty acid); Beta-elemen, phytol and N-[4-bromo-n-butyl]-piperidinone.

**Table 8:** Phytochemical compounds identified in the methanol leaf extracts of *Jacaranda mimosifolia*.

Peak#	RT	Area%	Name of compound	Molecular formula	MW	Nature of compound	Pharmacological activities
1	8.331	1.91	2-Nonadecanone 2, 4 dinitrophenyl hydrazine	C_25_H_42_N_4_O_4_	462	Nitrogen compound	*Antimicrobial
2	10.101	0.36	Ethyl iso-allocholate	C_26_H_44_O_5_	436	Steroid	*Antimicrobial, Anti-inflammatory, Diuretic
3	10.898	0.30	4-(3-hydroxy-1-propenyl)-2-methoxy-phenol,	C_10_H_12_O_3_	180	Phenolic compound	*Antimicrobial, Antioxidant, Anti-inflammatory
4	11.487	1.07	Hexadecanoic acid	C_16_H_32_O_2_	256	Fatty acid	Anti-inflammatory [100], Antioxidant, hypocholesterolemic nematicide, pesticide, anti androgenic flavor, hemolytic, 5-Alpha reductase inhibitor [101], potent mosquito larvicide [102].
5	12.869	2.09	1-β-d-Ribofuranosyl-3-[5-tetraazolyl]-1,2,4-triazole	C_8_H_12_N_4_O_5_	269		*Analgesics, antipyretics, anti-convulsants, anti-inflammatory, immune modulatory activity [103]
6	14.207	0.13	2,4-bis(1,1-dimethylethyl)-phenol	C_14_H_22_O	206	Phenol	*Antidiabetic, hepatoprotective, antimicrobial, antioxidant
7	14.954	0.31	Z,Z-4,16-Octadecadien-1-ol acetate	C_2_0H_36_O_2_	308	Acetate compound	No reported activity
8	15.747	0.21	3-Pyridinol	C_5_H_5_NO	95		No reported activity
9	17.325	0.37	Hexadeca-9-en-1-ol	C_16_H_32_O	240	Phenolic & Alcoholic compounds	*Antimicrobial
10	17.738	0.77	1-methyl-2-(3-methylpentyl)-cyclopropane	C_10_H_20_	140		No reported activity
11	18.252	1.21	Hexadecanoic acid, methyl ester	C_17_H_34_O_2_	270.4	Esters	Flavor Methyl palmitate has anti-inflammatory and anti-fibrotic effect. It prevents lung inflammation and fibrosis in rats
12	21.025	0.24	9,12-octadecadienoic acid(Z,Z)	C_18_H_32_O_2_	280	Fatty acid, Linolenic acid, methyl ester	Anti-inflammatory, Hypocholesterolemic, Cancer preventive, Hepatoprotective, Nematicide, Insectifuge, Antihistaminic, Antieczemic, Antiacne, 5-Alpha Reductase inhibitor, Antiandrogenic, Antiarthritic, Anticoronary, Insectifuge [104]
13	22.996	0.04	Beta-elemene	C_15_H_24_	204.35	Monocyclic sesquiterpenoid polyalkene	Anti-inflammatory and antitumor properties
14	23.2	0.21	Phytol	C_20_H_40_O	296	Diterpene alcohol	It produces anxiolytic and sedative effects. Antischistosomal [105] antioxidant [106], Antimicrobialmactivity [100] component of chlorophyll and vitamin E and K
15	23.45	0.07	Piperidinone, N-[4-bromo-n-butyl]-	C_9_H_16_BrNO	233	Alkaloid	*Antimicrobial Anti-inflammatory

RT = retention time; MW = molecular weight

* The biological activities are based on Dr. Duke’s phytochemical and botanical databases [107].

The biological activities listed in Table 8 are based on Dr. Duke’s phytochemical and botanical databases by Dr. Jim Duke of the Agricultural Research Service/USDA.

### 4. Conclusion

In this study, we showed that *Jacaranda mimosifolia* extracts and their fractions demonstrated promising antioxidant, anti-inflammatory, anti-microbial and anti-cancer properties, particularly for ME and CF. These biological activities were tentatively ascribed in part to the polyphenolics as well as other constituents of these fractions. Overall, the results obtained provide a biochemical rationale for further chemical analysis as well as animal and clinical studies with an antioxidant and chemotherapeutic focus.

## Supporting information

S1 Fig (TIFF)

## Abbreviations

NDGA: Nordihydroguaiaretic acid
DMSO: Dimethyl sulfoxide
GAE: Gallic acid; QE, Quercetin
DPPH: 2,2-diphenyl-1-picrylhydrazyl
EDTA: Ethylenediaminetetraacetic acid
TCA: Trichloroacetic acid
TBA: Thiobarbituric acid
NBT: Nitro blue tetrazolium chloride
PMS: Phenazine methosulfate
SDA: Sabouraud dextrose agar
MIC: Minimal inhibitory concentration
DPPH: 2,2-diphenyl-1-picrylhydrazyl
SRB: sulforhodamine B dye
LSD: Least significant difference
