# Supplementary figures and images for: GC-MS analysis, antimicrobial, antioxidant, antilipoxygenase and cytotoxic activities of Jacaranda mimosifolia methanol leaf extracts and fractions
Source: PLoS One. 2020 Jul 29;15(7):e0236319. doi: 10.1371/journal.pone.0236319 (PMC7390342; doi:10.1371/journal.pone.0236319)

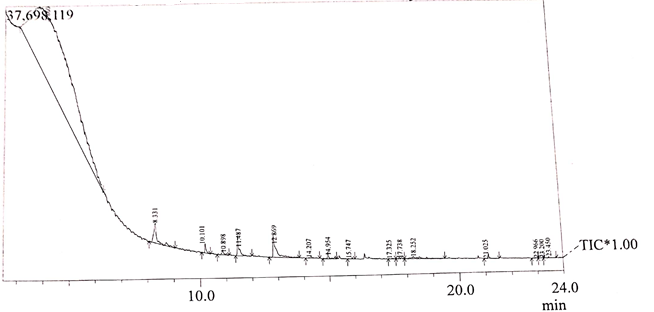

Supplement: S1 Fig — (TIFF) [file pone.0236319.s001.tiff]
